# Physical and chemical controls on habitats for life in the deep subsurface beneath continents and ice

**DOI:** 10.1098/rsta.2014.0293

**Published:** 2016-01-28

**Authors:** John Parnell, Sean McMahon

**Affiliations:** 1 School of Geosciences, University of Aberdeen, Aberdeen AB24 3UE, UK; 2 Department of Geology and Geophysics, Yale University, New Haven, CT 06511, USA

**Keywords:** deep biosphere, subglacial life, Antarctica, biogeochemistry, cell density

## Abstract

The distribution of life in the continental subsurface is likely controlled by a range of physical and chemical factors. The fundamental requirements are for space to live, carbon for biomass and energy for metabolic activity. These are inter-related, such that adequate permeability is required to maintain a supply of nutrients, and facies interfaces invite colonization by juxtaposing porous habitats with nutrient-rich mudrocks. Viable communities extend to several kilometres depth, diminishing downwards with decreasing porosity. Carbon is contributed by recycling of organic matter originally fixed by photosynthesis, and chemoautotrophy using crustal carbon dioxide and methane. In the shallow crust, the recycled component predominates, as processed kerogen or hydrocarbons, but abiotic carbon sources may be significant in deeper, metamorphosed crust. Hydrogen to fuel chemosynthesis is available from radiolysis, mechanical deformation and mineral alteration. Activity in the subcontinental deep biosphere can be traced through the geological record back to the Precambrian. Before the colonization of the Earth's surface by land plants, a geologically recent event, subsurface life probably dominated the planet's biomass. In regions of thick ice sheets the base of the ice sheet, where liquid water is stable and a sediment layer is created by glacial erosion, can be regarded as a deep biosphere habitat. This environment may be rich in dissolved organic carbon and nutrients accumulated from dissolving ice, and from weathering of the bedrock and the sediment layer.

## 1. Introduction

**Figure 1.**
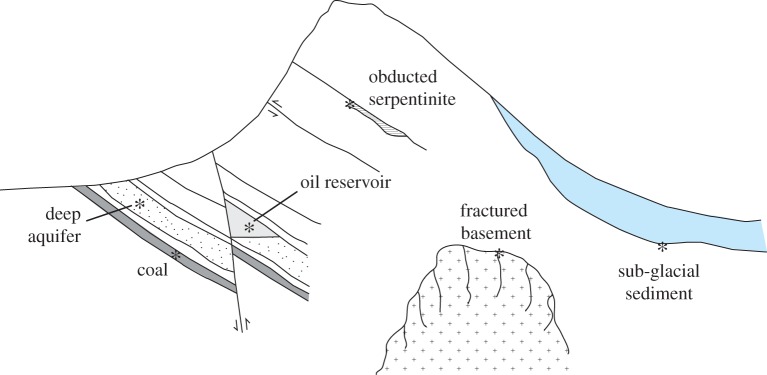
Schematic diversity of settings for deep biosphere activity in continental regions. (Online version in colour.)

There is abundant evidence that a substantial proportion of life on Earth resides in a subsurface deep biosphere. Cores and water samples from beneath both the continents and the ocean floor yield microbial (bacterial, archaeal and fungal) populations, which microbiological and genetic studies show are distinct from those at the surface, and thus represent life from a separate habitat rather than a contamination [1,2]. The continental deep biosphere (figure 1) conventionally includes microbes active in subsurface aquifers and fracture systems, but can also be extended to include microbes beneath ice sheets [3]. Its total biomass is likely of the order of 10^17^ g C, and may well exceed that of microbes in marine sediments [4]. Despite its abundance, the requirements to support life in the subsurface place major constraints on its distribution and activity, with starvation conditions the norm and generation times likely measured in the thousands of years [5]. This paper outlines the requirements for subsurface life and considers the particular case of the subglacial deep biosphere.

## 2. Requirements for life in the subsurface

The fundamental requirements of life are liquid water, space in which to live, carbon and a suite of other nutrients to make and replenish biomass, energy for growth and maintenance, and protection from physical and chemical stressors (extreme temperature, salinity, etc.). Whereas the inter-related requirements for space, energy and carbon are abundantly met by much of the Earth's surface, they can be severely limiting in the subsurface, with the result that biomass declines rapidly with depth in the uppermost kilometre [4]. The variability in subsurface chemical environments must also cause high diversity in growth and biomass. It should be remarked that microbial productivity is unlikely to be limited by confining pressure in any natural environment, because prokaryotic cells have been shown to survive and reproduce at pressures far higher than those encountered in the Earth's habitable crust [6]. The ultimate constraint on the depth of biological penetration in the crust is probably temperature, which increases with depth as a function of the local heat flux and thermal conductivity. In the laboratory, the maximum temperature seen to allow microbial reproduction is 122°C [7]. Temperatures typically exceed this limit at depths of around 5 km on land. The deepest living organisms yet recovered (to our knowledge) from continental boreholes hailed from depths of 3.6 km (48°C) and 5.3 km (70°C), respectively, while another deep borehole failed to detect life at 4 km depth (110°C) [8,9]. However, the measurement and sampling of deep systems is, to date, very limited. In addition, hydrothermal systems bring high temperatures to the surface, and thereby allow thermophiles to occupy porous habitats.

**Figure 2.**
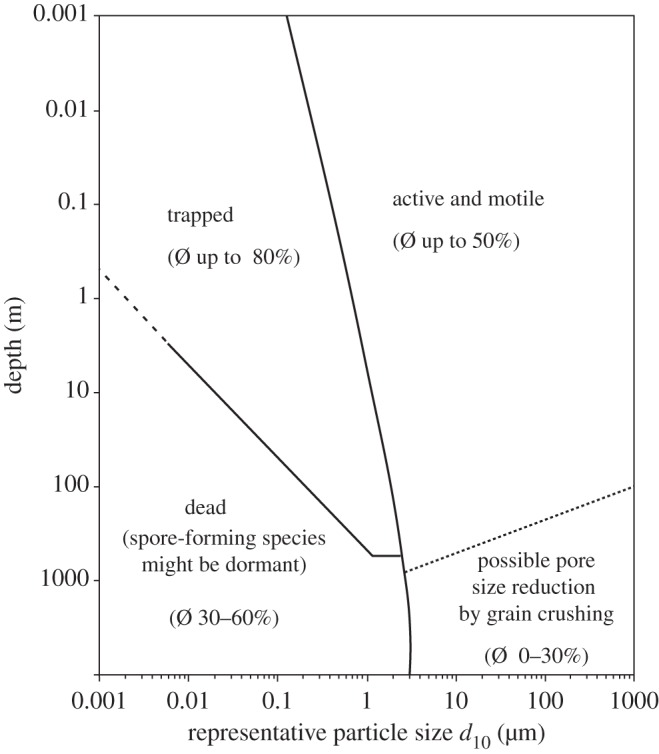
Fields of activity for microbial life, according to particle size and depth (adapted from [12]), indicating range of porosities in each field.

In the continental subsurface, living space is provided by the porosity (void space) of rock, sediment and ice. In the basal layers of ice sheets several kilometres thick, living space is available in the thin veins of liquid water on ice-crystal boundaries [10]. The uppermost part of the continental sediment column and crust is highly porous and commonly aerated, forming a ‘vadose zone’ that in arid zones reaches a few hundred metres in depth. Within this zone, microbes live in attachment to grain surfaces [11]. Even below the water table, cells attached to particle surfaces have been found to outnumber those suspended in groundwater by two to three orders of magnitude [4]. The specific surface area of the substrate may therefore also be an important control on subsurface cell densities. The constraints of particle size and related porosity can be used to allocate domains at progressive depths (figure 2) in which microbes can be alternatively active and motile, trapped or dead [12].

In rocks and sediments, the decay of porosity with increasing depth is on average approximately exponential, but varies strongly with the local rock or sediment type and geological history. Although clastic sedimentary rocks generally have higher primary porosities than crystalline rocks, poorly sorted or highly cemented sediments contain little porosity, whereas vesicular volcanic rocks are commonly highly porous. Igneous and metamorphic rocks also have higher compressive strengths and therefore retain their porosity at greater burial depths [13]. Porosity in all rock types may be increased secondarily at depth by pressure dissolution of minerals. On the other hand, fluid circulation can encourage the closure and loss of pore space by mineral precipitation. For example, pore-filling sulfides precipitated by microbial activity can lead to a loss of porosity and habitat [14].

Carbon, like nitrogen, phosphorus and a host of minor nutrients, is required for the construction and repair of microbial cells and spores. In the subsurface, carbon is contributed by the recycling of organic matter originally fixed by photosynthesis at the surface, and by crustal carbon dioxide and methane. In the shallow continental crust, the recycled component predominates, as kerogen (ancient organic matter), bitumen and dissolved organic carbon (DOC). Even deeply buried hydrocarbons continue to sustain life: onshore oil and gas reservoirs are commonly biodegraded and have yielded culturable anaerobic heterotrophs from several kilometres depth [15]. Microorganisms can directly oxidize the kerogen in shale [16]. The thermal maturation of organic-rich mudrocks also releases CO_2_, methane, acetate and other molecules from which microbes can obtain carbon [17,18]. However, in the crystalline continental basement, organic matter is relatively scarce. Here, autotrophy may therefore outstrip heterotrophy. Several abiotic geological processes can supply inorganic carbon. Hydrothermal activity, magmatic degassing and the thermal metamorphism of carbonates produce both CO_2_ and methane [19,20]. Low-temperature water–rock reactions in the presence of CO_2_ also generate methane, notably the serpentinization of mafic and ultramafic igneous rocks, e.g. in ophiolites [21].

Microbes obtain energy via redox reactions; in most metabolic systems, the transfer of electrons from reductants to oxidants is coupled to the shuttling of protons across a membrane, driving adenosine triphosphate synthesis. Although numerous redox couples are employed in the subsurface, many are in meagre supply and most yield little energy compared with photosynthesis and aerobic respiration. Energy limitation is consequently thought to be a widespread constraint on the productivity of the deep biosphere [5]. It seems likely that the oxidation, both aerobic and anaerobic (including fermentation), of organic matter originally derived from photosynthesis accounts for the greater part of subsurface productivity, supplemented by chemolithoautotrophy.

Biologically important oxidants in the crust include atmospheric O_2_, sulfate and nitrate, all of which fuel both heterotrophic and autotrophic metabolisms. Near the surface, molecular oxygen is rapidly consumed by the oxidation of organic matter, leaving anaerobic sulfate reduction, methanogenesis and acetogenesis to dominate in deeper environments. Electron acceptors indigenous to the subsurface include magmatic/hydrothermal CO_2_ and Mn(IV) and Fe(III) in minerals [22,23]. The thermal degradation of deeply buried organic matter also produces CO_2_ as well as a range of electron donors (including H_2_, H_2_S and methane) [17,18]. Alternative electron donors in the crust include sulfur, nitrite and reducing gases (e.g. H_2_, CH_4_, 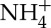 and HS^−^) derived from the mantle, from hydrothermal and metamorphic fluids, and from low-temperature water–rock reactions (notably serpentinization). Other mechanisms potentially capable of producing biologically significant concentrations of H_2_ in the subsurface include the radiolysis of pore water by radioactive decay, and ‘mechanoradical’ chemistry associated with earthquakes [24,25]. Despite the consumption of oxygen near-surface, deep environments need not be anoxic, as they can be penetrated by surface-derived oxidizing waters, or oxidized by alternative chemical processes.

Microbes have also been shown to oxidize a range of divalent cations in rocks and minerals [26]. The importance of substrate mineralogy is therefore (at least) threefold: specific minerals represent effective chemical energy sources, adsorption sites for organic compounds and surface attachment sites for microbial cells, with widely varying specific surface areas.

The requirements for carbon, energy and space are inter-related in this and numerous other ways. Burial depth strongly controls all three: as we have seen, carbon and energy are consumed in the shallow crust but potentially generated by other means at greater depths; likewise, the generation of secondary porosity by mineral dissolution may offset the decline in primary porosity caused by pressure compaction. Organic compounds provide chemical energy as well as carbon for biomass, and their supply and distribution, as well as those of other nutrients, depend on the amount and configuration of porosity. To allow water, cells and metabolites to circulate, pore spaces must be connected together laterally and vertically. The importance of adequate permeability is exemplified in a study by Hoehler & Jørgensen [5] which showed that without water flow to replenish sulfate levels in marine sediment, the energy that could be derived from sulfate reduction was insufficient to power the movement of flagellae that would allow microbial movement. Like porosity, this property of permeability or hydraulic connectivity also varies substantially between different geological substrates and with the pervasiveness of faulting, fracturing, mineralization and pressure dissolution. Lithological boundaries may provide the optimal balance of conditions for subsurface habitability. For example, at mudrock–sandstone interfaces, nutrients can diffuse from the low-porosity mudrock into the relatively porous but nutrient-poorer sandstone [27].

## 3. The geological record

The deep biosphere represents a major component of life on Earth. It is, therefore, likely to have persisted through much of geological history, and indeed some workers propose that life had a thermophilic, subsurface origin. Several types of evidence support a long-lived deep biosphere, including carbonate concretions in mudrocks; sulfides precipitated in mudrocks, at mudrock–sandstone interfaces and at redox boundaries in sandstone aquifers; sulfides in serpentinites and vesicular basalts; bioalteration features in volcanic glass; and mineralized filaments in hydrothermal and other fracture systems. In several cases, this evidence extends back to a Precambrian record [27,28,29]. This long-term geological record has a profound implication for the nature of life on Earth. Today, about half of the Earth's biomass is in the deep biosphere [4,30]. Most of the surface biomass is accounted for by land plants. However, land plants are a relatively young component of life on Earth, only prevalent for 10% of the planet's history. Before land plants, the continents were encrusted with microbial mats of much lower biomass. The productivity of these mats may have pumped carbon into the subsurface in the same way that low-biomass, high-productivity marine phytoplankton fuel today's deep marine biosphere. If the size of the deep biosphere has been of constant order of magnitude for billions of years, for most of that time the large majority of life on Earth has been in the subsurface. Expressed another way, the Earth is characterized by a subsurface habitat for life, and as Earth is the only example available we might infer that life on other planets is most likely to be subsurface. During periods of global glaciation in Earth's history, such as the so-called ‘Snowball Earth’ episodes in the Neoproterozoic, the subsurface habitat would likely have included sub-ice environments [31,32,33,34], although this remains to be proved.

## 4. The special case of the sub-ice deep biosphere

**Figure 3.**
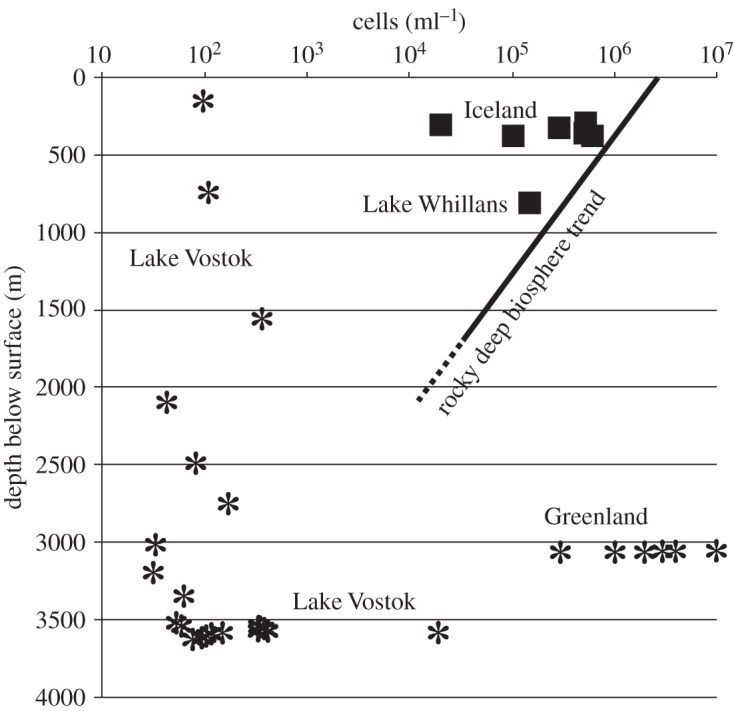
*In situ* subglacial cell counts measured in the ice above Antarctic subglacial Lake Vostok, in Antarctic subglacial Lake Whillans, in two subglacial lakes in Iceland and in the GISP2 ice core in Greenland. Asterisk symbols indicate measurements taken from ice samples. Box symbols indicate measurements taken from subglacial liquid water. The trend shown is suggested by the distribution of cell counts in continental aquifers [4,38,39,41,42,43,79,80,81].

There is much evidence for microbial activity in subglacial settings [32,35,36,37,38]. Direct counts of deep sub-ice microbial cells have been made in Antarctica, Greenland and Iceland (figure 3 and table 1). The subglacial Lake Vostok in Antarctica has been found to support prokaryotic cell counts of the order of 10^4^ per millilitre of water at 3.6 km depth [39]. These microbes are inferred to use oxygen and nitrate as electron acceptors, supplied by the melting of basal ice, as well as sulfate derived from sulfide-mineral weathering [40]. Silty ice recovered from a depth of approximately 3050 m in a borehole in the Greenland ice sheet contains population densities up to 10^7^ cells per millilitre in the ice and up to 10^10^ attached cells per cubic centimetre of silt [41]. These organisms include Fe(III)-reducers attached to clay grains, as well as methanogens. Two subglacial lakes in Iceland have been found to contain diverse populations of bacteria. Grímsvötn Lake yields bacterial cell counts of approximately 10^4^ per millilitre under 300 m of ice [42]; Skaftárketill Lake yields up to 5×10^5^ bacterial cells per millilitre [43]. High cell densities have been found in subglacial sediments in several other regions, including the Alps [44], New Zealand [45], the Canadian High Arctic [46], Svalbard [47], the Antarctic Peninsula [48] and the West Antarctic Ice Sheet [37].

**Table 1.** Intraglacial and subglacial cell counts plotted in figure 3. Depths in ice are rounded to the nearest 10 m.

locality	substrate	depth (m)	mean cell count (ml^−1^)	references
GISP2, Greenland	ice core	3040–3050	3.5×10^6^	[41]
Grímsvötn Lake, Iceland	subglacial lake	300 (ice)+10 (lake)	2.1×10^4^	[42]
Western Skaftá Lake, Iceland	subglacial lake	300 (ice)+112 (lake)	5.0×10^5^	[43]
Lake Skaftárketill, Iceland	subglacial lake	280–390	4.6×10^5^	[79]
Lake Vostok, Antarctica	ice core	3590	1.9×10^4^	[80]
		180–3570	1.7×10^2^	[81]
		3520–3620	1.9×10^2^	[39]
Lake Whillans, Antarctica	subglacial lake	800 (ice)+<2 (lake)	1.3×10^5^	[38]

**Figure 4.**
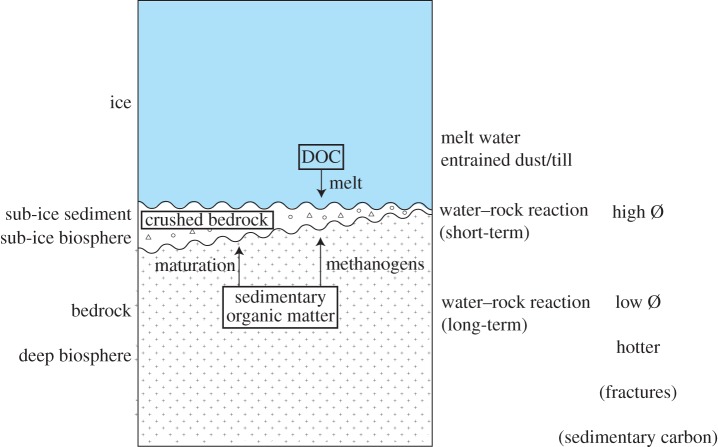
Schematic profile of ice above sub-ice and deep bedrock biospheres. Sub-ice biosphere is characterized by high porosity and short-term water–rock interaction. Bedrock biosphere is characterized by lower porosity and long-term water–rock interaction. Boxed terms indicate potential sources of carbon to sub-ice biosphere (DOC in ice, crushing of bedrock, maturation of sedimentary organic matter, methanogenesis using sedimentary organic matter). (Online version in colour.)

Most of this activity depends on resources derived from minerals and organic matter in sediments below the ice, including large sedimentary basins [33,49]. High temperature is not a likely constraint, and low temperature is effectively limited by liquid water. Critically, there is evidence for widespread liquid water below ice sheets [50,51], which allows microbial activity to persist under both oxic and anoxic conditions [33]. Sub-ice microbial activity occurs especially in the sediment layer created by glacial erosion, but also in more evolved environments such as subglacial lakes. There is a contrast between this sub-ice deep biosphere and the bedrock deep biosphere below (figure 4). The sub-ice sediment may have high porosity, commonly at least 10% and up to 40% [52], but the porosity is likely to be much more depleted in the bedrock. The high porosity, and a high nutrient flux (see below), accommodate high cell densities in the sub-ice sediment [44,45]. The bedrock below is hotter, and in some cases may have fracture porosity, both of which are favourable to life, but even where fractured the rate of water flow and nutrient supply will not approach the high rates pertaining in the sub-ice sediment [50]. The sub-ice deep biosphere is distinctive in both the materials and processes that contribute resources. In particular, the melting of ice and the mechanical grinding of bedrock are potentially significant sources of carbon and nutrients that do not contribute to the endolithic subcontinental biosphere.

Where ice has been progressively buried from the surface down to the base of an ice sheet, it carries components introduced at the surface. The environmental records contained within ice cores are evidence of this stored material. The entrained components become released on melting of the ice. These are dominated by wind-borne components. Dust (visible particles mostly smaller than sand) from abrasion of bedrock would in most cases be from sources within tens of kilometres. This could include nutrients depending on the nature of the bedrock, and also organic compounds [53]. Finer aerosols (colloidal fine particles and liquid droplets) introduce material from much further afield, globally in the case of large volcanic eruptions. In Antarctica, the range of sources includes the Antarctic continent, the surrounding ocean, continents beyond the ocean and continental shelf exposed during glacial stages [54,55]. Aerosols contribute to ice-crystal nucleation, which aids their precipitation to the ground surface. A study of amino acids in Antarctic aerosols [56] illustrates several key points. Amino acids derived from marine life in the surrounding seas become entrained in aerosols through bubble bursting on the ocean surface. The aerosols are then transported over the Antarctic continent for several days before descending onto the ice sheet [57]. Organic compounds may be created or destroyed during transport by photochemical processes. Coagulation of particles also occurs, but the amino acids are concentrated in the sub-micrometre fraction. In the case of amino acids, they contribute nitrogen as well as carbon to the ice, adding to other nitrogen-bearing components in aerosols such as nitrate and ammonium ions [58].

**Figure 5.**
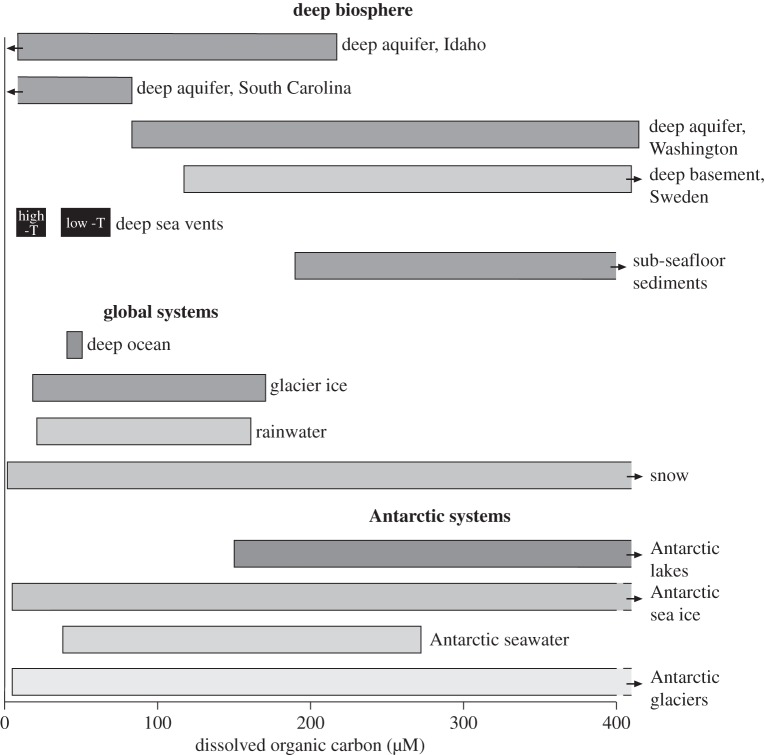
DOC levels measured in a range of settings, including global media, deep aquifers and Antarctic environments (data from [59,60,61,62,63,64,65,66,82,83]).

An indication of the potential magnitude of the sub-ice and other polar biospheres is given by DOC levels in Antarctic media. DOC levels in Antarctic lakes, glacier ice and sea ice all range up to values exceeding 400 μM [59,60]. These values are higher than those typically encountered in deep aquifer waters that support subsurface microbial communities [61,62,63], and also in globally widespread media such as rainwater, deep ocean water and sub-seafloor sediments [64,65,66] (figure 5). However, inferred values for biomass per cell of 11 fg C from the McMurdo Dry Valley lakes, Antarctica [67] and 19 fg C from the Greenland ice sheet [68] are below the mean 26 fg C typical for cells in starvation conditions [69], suggesting that nutrient availability in the polar environments is limited. Despite large uncertainties in data, attempts have been made to estimate the biomass in the sub-ice biosphere [31,70]. This effectively means the biomass in the sub-ice aquifers, as the biomass in ice sheets and subglacial lakes is negligible by comparison [70]. The most recent estimate [70] suggests about 4.4 Pg C biomass, comparing with an estimated 14–135 Pg C biomass in the subcontinental biosphere [4]. If the same mean biomass per cell was used, the two estimates would be very close. Input parameters of 1 km sub-ice aquifer depth and 2×10^7^ cells g^−1^ throughout may be reduced as more data become available, but the estimate for sub-ice life is high enough to suggest that during episodes of global glaciation, life in this setting could predominate if much of the subglacial environment remains unfrozen.

The special case of mechanical grinding of bedrock at the base of glaciers can accelerate the release of nutrients and energy sources, including iron, phosphorus and sulfides, through formation of fresh mineral surfaces with large surface area. Rock flour in particular presents very high surface area per unit mass. Subglacial microbial activity includes sulfide oxidation, Fe(II) oxidation, sulfate reduction, nitrate reduction, Fe(III) reduction, Mn(IV) reduction, aerobic respiration using oxygen from ice melt, and the release of carbon dioxide to weather silicates and carbonates [33,35,49]. Subglacial meltwaters are consistently rich in dissolved sulfate, implying that it has been liberated from the weathering of pyrite in bedrock and sediment by oxidation [71] and microbes that particularly alter pyrite as they can use both iron and sulfur through redox reactions [34]. Pyrite, therefore, may be the predominant mineralogical control on subglacial microbial community structure [34]. Microbial activity can also release gases that are energy sources for microbes, although direct measurements of activity have yet to be made. In addition to the methane produced by microbial processing of sedimentary organic matter [35], methane generated or trapped in bedrock can be released by crushing, as found in other environments [72]. Detritus from crystalline bedrock, known to yield hydrogen to groundwaters [73], can also release hydrogen on crushing [72], which fuels chemosynthesis. Given the widespread erosion beneath ice sheets, the supply of hydrogen may be a significant and as yet unrecognized driver for subglacial microbial activity.

Below both the West Antarctic Ice Sheet and Greenland Ice Sheet, there are proportionally large areas of high heat flow. This causes relatively high rates of basal ice melting [74,75], which may enhance nutrient availability by weathering below the ice sheets. Volcanic heat maintains subglacial lakes in Iceland, and hydrothermal carbon dioxide contributes to the carbon budget there [43]. It is similarly speculated that hydrothermal gases are contributed to subglacial environments in the West Antarctic [76]. A significant proportion of thermotolerant species in Lake Vostok accretion ice is consistent with geothermal activity [77]. Active volcanism would leave ash layers in the ice, as recorded in Marie Byrd Land, Antarctica [78], which would eventually feed nitrogen, phosphorus and other nutrients to the subglacial environment. The high heat flow is also likely to enhance the main rock-hosted deep biosphere beneath. Higher temperatures promote increased rates of (bio)geochemical reactions, the convection of water to replenish nutrients and the geological processes generating the heat could also cause fracturing to further enhance permeability and porosity.

## 5. Conclusion

The subsurface biosphere is, proportionally, a very significant component of life on Earth. Several factors contribute to this, including connected pore space down to several kilometres depth, widespread groundwater flow, heat-assisted mineral alteration which provides nutrients and energy, and a supply of carbon. The sub-ice biosphere takes advantage of high porosity and water flow, and the availability of nutrients/energy from fresh erosion products. Carbon is available from the activity of methanogens, methane released by crushing of bedrock and the direct access to sedimentary organic matter including DOC from melting ice. The crushing of bedrock should additionally yield hydrogen to allow the possibility of subglacial chemosynthesis. Future exploration should take us from speculation to quantification of these subsurface processes.
